# PI3K/AKT-mediated upregulation of WDR5 promotes colorectal cancer metastasis by directly targeting ZNF407

**DOI:** 10.1038/cddis.2017.111

**Published:** 2017-03-16

**Authors:** Xin Tan, Shuai Chen, Jiangxue Wu, Jiaxin Lin, Changchuan Pan, Xiaofang Ying, Zhizhong Pan, Lin Qiu, Ranyi Liu, Rong Geng, Wenlin Huang

**Affiliations:** 1 State Key Laboratory of Oncology in South China, Guangzhou 510060, PR China; 2 Collaborative Innovation Center for Cancer Medicine, Sun Yat-Sen University Cancer Center, Guangzhou 510060, PR China; 3 Guangdong Lung Cancer Institute, Guangdong General Hospital & Guangdong Academy of Medical Sciences, Guangzhou 510080, PR China; 4 Medical Oncology, Sichuan Cancer Hospital and Institute, Second People's Hospital of Sichuan Province, Chengdu 614000, PR China; 5 Department of Radiation Oncology, Hubei Cancer Hospital, Wuhan 430079, PR China

## Abstract

Colorectal cancer (CRC) is the third most common cause of cancer deaths, and has a high rate of liver and lung metastasis. Unfortunately, distant metastasis is the main barrier for advanced CRC therapy and leads to a very low survival rate. In this study, we identified WDR5, a vital factor that regulates vertebrate development and cell self-renewal and reprogramming, as a novel prognostic marker and therapeutic target for CRC patients. We demonstrate that WDR5 is upregulated in CRC tissues and promotes CRC metastasis both *in vitro* and *in vivo*. In an effort to investigate the impact of WDR5 on CRC cell fate, we treated CRC cells with growth factor and inhibitor. We report that WDR5 is a novel factor in the metastasis of CRC by triggering epithelial–mesenchymal transition (EMT) process in response to the PI3K/AKT signaling pathway. Moreover, WDR5 shows a direct binding to the ZNF407 promoter on regulating cellular EMT process, leading to CRC metastasis. Hence, our findings strongly position WDR5 as a valuable marker for CRC, and inhibiting WDR5 or the associated signaling pathways may be an effective strategy for the future development of anti-CRC therapy.

Colorectal cancer (CRC) occupies the third place in the cancer death, and is common for both men and women worldwide.^1, 2^ The incidence rate has not significantly changed in the past decade and has even increased among adults younger than 50 during this period.^2^ Although new screening strategies and improvements in treatment continue to emerge, the prognosis differs widely.^3, 4, 5^ Moreover, distant metastasis remains the main barrier for CRC therapy.^6^ Thus, it is imperative to discover molecular mechanisms and genetic alterations and to explore new biomarker and therapeutic targets for CRC.^7^

Studies confirmed that the MLL and hCOMPASS complexes regulate global H3K4 methylation,^8^ and one of the components of these complexes, MLL1,^9^ has been reported to have oncogenic roles by modulating chromatin structures. WDR5 is the most highly conserved subunit across all of the human COMPASS-related complexes,^10^ which generate H3K4me3 to influence cell development,^8, 11, 12^ especially vertebrate skeletal system development.^13, 14^ Recent studies reported that WDR5 is abnormally expressed in many cancers. A cohort study of leukemia patients suggested that WDR5 may have oncogenic effects in leukemogenesis.^15^ In addition, researches show that WDR5 has high expression level in prostate cancer, bladder cancer, and breast cancer, and has a positive correlationship with advanced stage and poor survival.^16, 17, 18^ It is well established that CRC may share some similarities with these other types of cancer. In addition, WDR5 mediates somatic cell reprogramming and embryonic cell self-renewal, while epithelial–mesenchymal transition (EMT) was reported to participate in the achievement of stemness as a key step.^19, 20^ Therefore, these achievements suggest that WDR5 might have an oncogenic role to play in the early steps of the invasion–metastasis cascade by regulating EMT to enable cancer cells to invade deep into the stroma, and then translocate to distant parenchyma.

The PI3K/AKT pathway is frequently observed to be hyperactivated in CRC caused by genetic aberrations, such as growth factors overexpression, gene mutations and so on. Recent studies have presented that PI3K/AKT pathway activation is a key character of EMT.^21^ One study in prostate cancer demonstrated a relationship between radioresistance and EMT via overactivating the PI3K/AKT/mTOR signaling pathway.^22^ Another study showed that activated AKT causes EMT based on the regulation of EMT-specific markers in human squamous cell carcinoma lines.^23^ Moreover, Riquelme E *et al.*^24^ advocated a methyltranferase, EZH2, was modulated by the PI3K/AKT transduction pathway in lung cancer. As WDR5 has great influence on assembly and enzyme activity of the MLL histone methyltransferase complex,^25, 26^ and the mechanism by which WDR5 levels are elevated in CRC is rarely known, we investigated the regulation of WDR5 in CRC.

In the present study, we discovered that high level of WDR5 expression in primary CRC is strongly correlated with poor prognosis. Both *in vitro* and *in vivo* assays indicated that WDR5 upregulation is sufficient to promote CRC metastasis. More importantly, molecular studies revealed that WDR5-transfected cells underwent EMT and were modulated by the PI3K/AKT signaling pathway. Furthermore, gene expression microarrays were used to identify ZNF407, which was reported to affect tumor progression,^27, 28^ as a novel target of WDR5, which markedly increased CRC cell migration. Thus, we propose that WDR5 is a promising target in CRC prognostics and therapeutics.

## Results

### WDR5 is frequently detected to be upregulated in CRC cell lines and tissues

**Figure 1:**
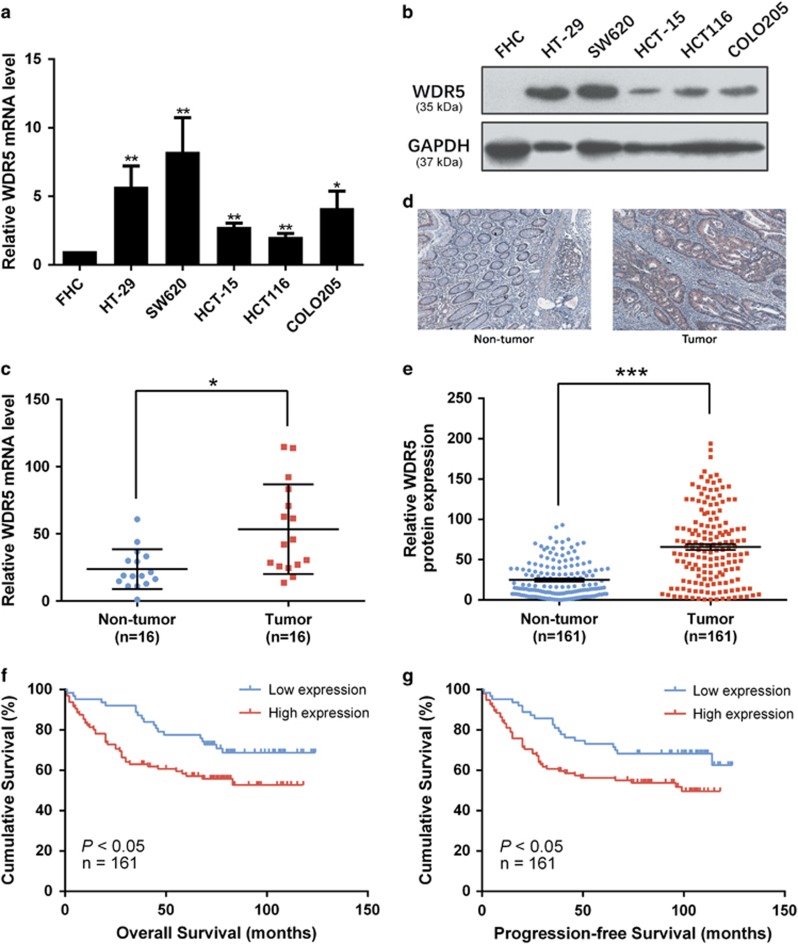
High levels of WDR5 are measured in CRC cells and tissues, and WDR5 serves as an independent prognostic factor for CRC metastasis. (**a**) Relative WDR5 mRNA expression normalized to GAPDH in five CRC cell lines and a normal colon epithelial cell line (qRT-PCR). Data are presented as the mean±S.D. of triplicate samples (*P<0.05; **P<0.01). (**b**) Western blot analysis of WDR5 expression in the indicated CRC cell lines. GAPDH was employed as a loading control. (**c**) Relative WDR5 mRNA expression levels in 16 pairs of CRC tissues (qRT-PCR; *n*=16; *P<0.05). (**d**) Representative IHC staining of WDR5 expression in matched primary CRC samples and corresponding non-tumor tissue (original magnification, × 200). (**e**) WDR5 protein expression in paired CRC and adjacent non-tumor tissues (IHC; *n*=161; ***P<0.001; independent *t*-test). (**f** and **g**) Overall survival (**f**) and progression-free survival (**g**) curves were generated by Kaplan–Meier analysis according to the WDR5 protein expression levels of 161 paired CRC and non-tumor tissues (*P*<0.05)

For the purpose of addressing the potential role of WDR5 in CRC development, WDR5 expression levels were measured in human CRC cell lines and tissues. Results showed that all five CRC cell lines (HT-29, SW620, HCT-15, HCT116, and COLO205) had higher WDR5 expression level than the normal intestinal epithelial cell line (FHC) at both the mRNA level (Figure 1a; *P*<0.05) and the protein level (Figure 1b). In addition, WDR5 mRNA expression was markedly higher in colorectal tumor tissues than those in paired adjacent normal mucosal tissues (qRT-PCR; *n*=16; Figure 1c; *P*<0.05). Moreover, similar results can be counted in large samples from TCGA Colorectal Adenocarcinoma (COADREAD) that project that about twofold greater WDR5 mRNA expression levels were calculated in human CRC tissues than in normal tissues (Supplementary Figure S1B; *P*<0.001). Further, to examine endogenous WDR5 protein expression, we performed immunohistochemistry (IHC) on histopathologically confirmed CRC tissue sections with paraffin-embedded (*n*=161; for more information, see Supplementary Table S1).Results showed a markedly higher level of WDR5 protein expression in tumor tissues than that compared to paired non-tumor mucosal tissues (Figure 1d and e,Supplementary Figure S1A; *P*<0.001).

### Elevated expression of WDR5 is associated with CRC metastasis and predicts poor prognosis

Then, the association between WDR5 expression and clinicopathological characters was analyzed. Data presented in Table 1 implied that high WDR5 expression was significantly correlated with TNM stage, lymph node status, and distant metastasis, but not with gender, age, location, size, grade, or CEA level. As shown in Figure 1f and g, patients with a high WDR5-expression signature had a shorter OS, likewise, high expression level of WDR5 was consistently associated with a reduced PFS by Kaplan–Meier analysis significantly (*P*<0.05). In addition, according to the data from TCGA data sets, the correlation between WDR5 expression level and the patients' survival was analyzed by defining two different follow-up times (5 years or extend to 28 January 2016). Both results showed a shorter overall survival (OS) in high WDR5 group significantly with *P*<0.05 (Supplementary Figure S1C). Also, the 5-year rate for high WDR5 level patients is 45.6%, compared to over 70% of low WDR5-level group at the fifth year (Supplementary Figure S1D).

Moreover, Cox proportional hazards regression analysis embodied that the WDR5 level was independently related to survival, which implicated WDR5 as an independent prognostic factor for PFS and OS (shown in Table 2). Thus, these analyses indicate that WDR5 upregulation is clinically relevant to CRC metastasis, and WDR5 is an independent prognostic factor in CRC patients.

### WDR5 enhances the CRC cell migration and invasion ability *in vitro*

**Figure 2:**
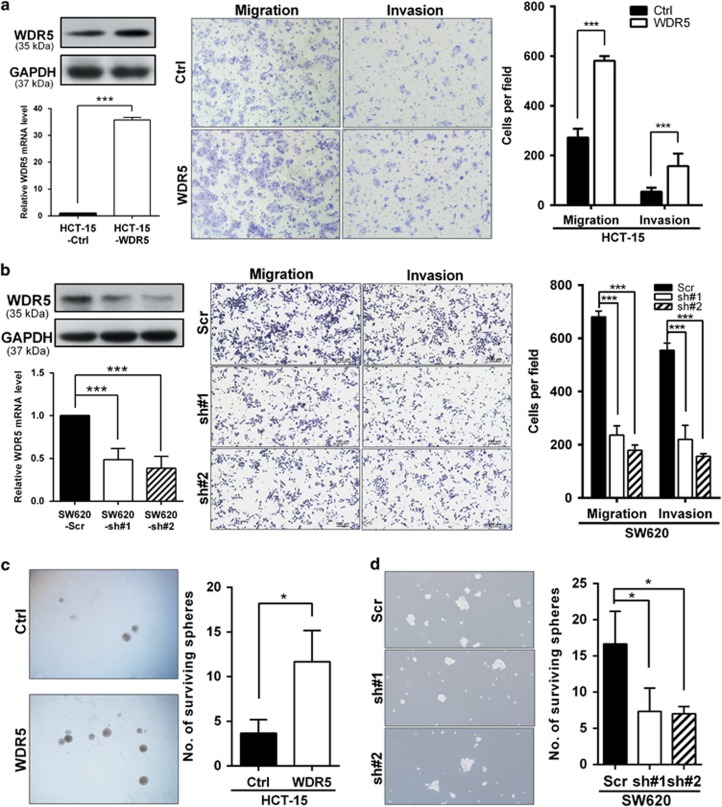
WDR5 promotes migration and invasion of CRC cells *in vitro*. (**a**) Left: qRT-PCR and western blot analysis of ectopic WDR5 expression in the HCT-15-Ctrl/WDR5 stable cell lines. Right: migration and invasion abilities were determined by transwell assays (***P<0.001). Representative images are shown. (**b**) Left: relative WDR5 mRNA and protein levels normalized to GAPDH levels in SW620-Scr/sh#1/sh#2 stable cell lines. Right: cell migration and invasion assays of SW620 were determined in the indicated SW620 cell lines as described. Representative images of cells are shown (***P<0.001). (**c**) Bright field micrographs showing the sphere formation ability of HCT-15-Ctrl/HCT-15-WDR5 stable cell lines. The number of surviving spheres was counted (*P<0.05). (**d**) Representative images of sphere formation by SW620-Scr/sh#1/sh#2 stable cell lines. The number of surviving spheres was counted (*P<0.05). All assays were reproduced in three times at least independently (independent Student's *t*-test)

By reason of the relationship between WDR5 and lymph node status and distant metastasis, the biological function of WDR5 in CRC cell migration and invasion was explored using transwell assays (with or without Matrigel) in the context of WDR5 overexpression or deletion. WDR5 expression was evaluated, and both migration and invasion were substantially enhanced when WDR5 was overexpressed in HCT-15 cells (Figure 2a; *P*<0.001). In contrast, downregulation of WDR5 sharply suppressed SW620 cell migration and invasion (Figure 2b; *P*<0.001).

Additional studies showed that overexpressing WDR5 with pcDNA3.1-WDR5 markedly promoted the motility and invasive ability of HCT116 cells, and siRNA-mediated knockdown of endogenous WDR5 expression strongly decreased HT-29 cell motility (Supplementary Figure S2; *P*<0.001).

Furthermore, to simulate the liquid nature of the circulatory system that is encountered during metastasis, we examined the sphere formation ability of CRC cells after altering WDR5 expression. Interestingly, HCT-15-WDR5 cells had increased capacity for sphere formation compared with HCT-15-Ctrl cells (Figure 2c; *P*<0.05). In contrast, the sphere-forming ability of SW620-sh#1 and SW620-sh#2 cells was significantly decreased (Figure 2d; *P*<0.05), suggesting that WDR5 maintains the vitality and stemness of CRC cells. Therefore, these results prove that WDR5 promotes the metastatic behavior of CRC *in vitro.*

### WDR5 promotes CRC metastasis in xenograft mouse model

**Figure 3:**
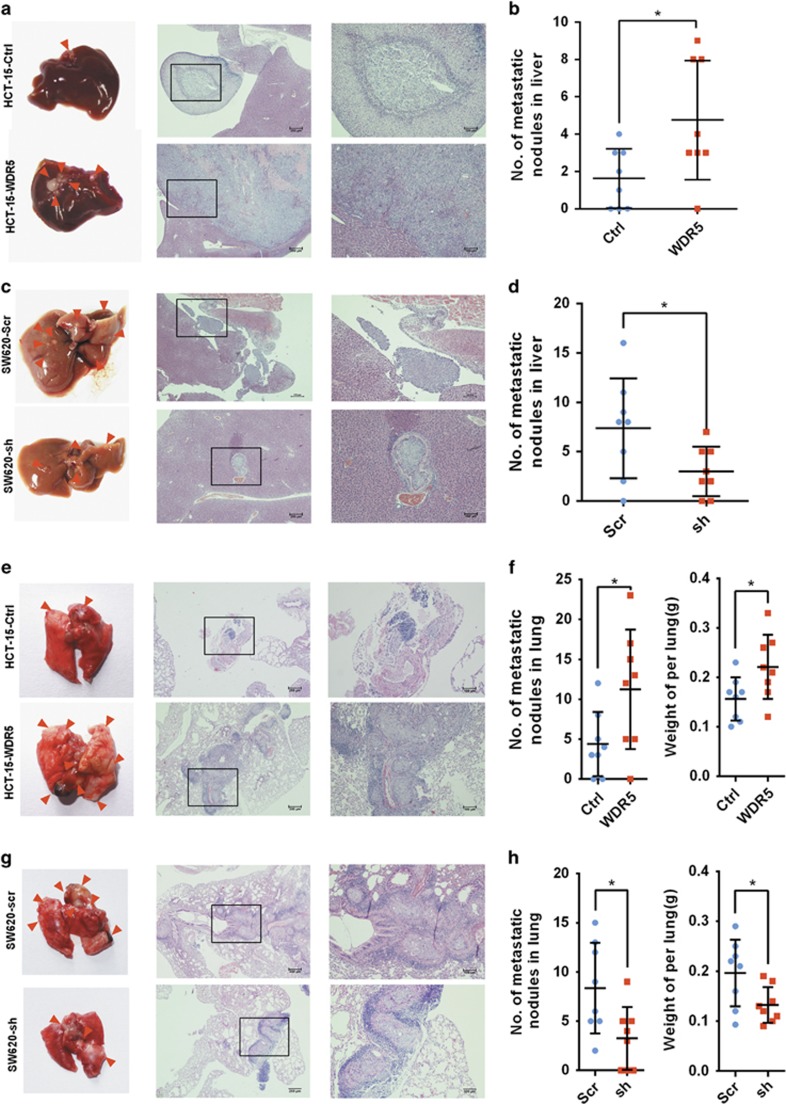
WDR5 enhanced the metastasis ability of CRC cells to the liver and lung in nude mouse models. (**a** and **c**) Hepatic metastasis model. Left: microscopic images of representative livers. The metastatic nodules are pointed out by arrows. Right: H&E staining of metastatic nodules in the liver. (**b** and **d**) Number of metastases per liver (*n*=8; *P<0.05). (**e** and **g**) Lung metastasis model. Left: microscopic appearance of representative lungs. Arrows indicate the metastasis nodules. Right: H&E staining of the metastatic nodules in the lungs. (**f** and **h**), number of metastases per lung (*n*=8; *P<0.05). Error bars: mean±S.D.

For liver metastasis model, HCT-15-WDR5 and HCT-15-Ctrl cell lines were injected into the spleens of nude mice. Most of the mice in the HCT-15-WDR5 group formed liver metastases, in contrast to those in the HCT-15-Ctrl group (Figure 3a and b; *P*<0.05). In addition, the metastases were larger in the HCT-15-WDR5 group than in the HCT-15-Ctrl group (Figure 3a, right).

Lung metastasis model *in vivo* was also developed. With H&E staining assessment, the HCT-15-WDR5 group showed more metastatic pulmonary nodules and increased lung weight compared with the HCT-15-Ctrl group (Figure 3e and f; *P*<0.05).

In addition, we utilized the *in vivo* liver and lung metastasis models with the SW620-sh#2 cell line, in which WDR5 was silenced, and the SW620-Scr cell line was a negative control. Livers and lungs were collected after 8 weeks, and the metastatic nodules on the surfaces were significantly smaller and fewer in number in the SW620-sh#2 group than in the SW620-Scr group (Figure 3c; *P*<0.05). Thus, these two models indicate that WDR5 promotes CRC metastasis *in vivo*.

### WDR5 promotes CRC cell motility by inducing EMT

**Figure 4:**
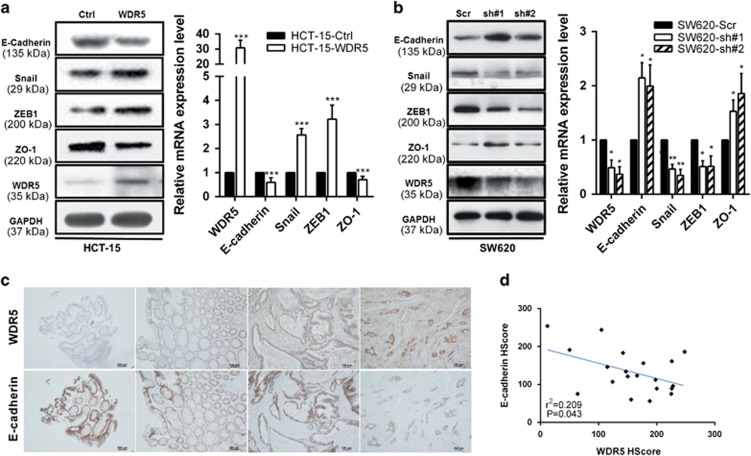
WDR5 induced epithelial–mesenchymal transition in CRC. (**a**) Indicated molecules were analyzed by western blot or qRT-PCR in HCT-15 cells stably overexpressing vector or WDR5. The data are presented as the mean±S.D. of triplicate samples (Student's *t*-test; ***P<0.001). (**b**) Indicated molecules were analyzed by western blot or qRT-PCR in HCT-15 cell lines with stable WDR5 knockdown. The data are presented as the mean±S.D. of triplicate samples (Student's *t*-test; *P<0.05; **P<0.01). (**c**) Representative IHC for WDR5 and E-cadherin in normal and CRC tissues. (**d**) A correlationship was detected between WDR5 and E-cadherin protein expression levels in 20 CRC tissues (Pearson's test, *n*=20, *r*=0.457, *r*^2^=0.209, *P*<0.05)

Given that EMT is a critical event involved in tumor invasion–metastasis cascade, the impact of WDR5 on EMT was analyzed based on the expression levels of EMT-specific proteins. Western blot analysis showed decreased E-cadherin and ZO-1 expression and increased expression of Snail1 and ZEB1 in WDR5-overexpressing cells compared with control cells. The qRT-PCR results further demonstrated that E-cadherin and ZO-1 were upregulated, while simultaneously, Snail1 and ZEB1 were downregulated at the mRNA level upon overexpressing WDR5 (Figure 4a). The opposite gene expression pattern was observed in the WDR5-silenced SW620-sh-cell lines (Figure 4b). The correlation between WDR5 and E-cadherin expression was further studied by IHC in the same cohort of 20 CRC samples. The results confirmed that E-cadherin expression had a significant negatively correlation with WDR5 expression (*r*^2^=0.209, *P*<0.05; Figure 4c and d). Overall, WDR5 regulates CRC metastasis by modulating epithelial- and mesenchymal-specific proteins.

### WDR5 mediates the EMT process regulated by the PI3K/AKT pathway

As *PIK3CA* and *PTEN* mutations are commonly detected in CRC, and hyperactivation of the PI3K/AKT pathway, which leads by these genetic aberrations, is frequently observed in CRC, which leads to reduced apoptosis, increased proliferation, and induced EMT process, we stimulated the PI3K/AKT pathway by treating cells with IGF-1 for 24 h and then evaluated the expression of certain proteins that are involved in the PI3K/AKT pathway and EMT.

**Figure 5:**
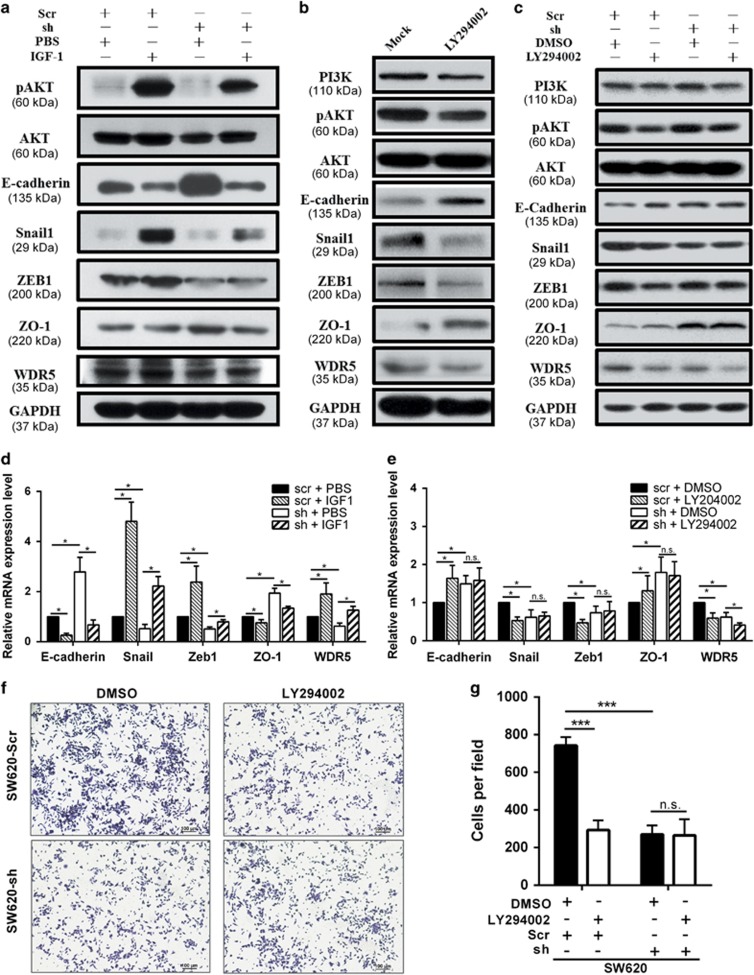
The PI3K/AKT pathway activates WDR5-induced EMT. (**a** and **d**) Western blotting (**a**) and qRT-PCR (**d**) were performed to measure the expression of WDR5 and EMT markers with IGF-1 (100 ng/ml) treated for 24 h (*P<0.05). (**b**) Western blotting demonstrated that the AKT inhibitor LY294002 (10 *μ*M) effectively decreased WDR5 expression and regulated EMT-associated genes. (**c** and **e**) Western blotting (**c**) and qRT-PCR (**e**) were conducted to measure the effect of LY294002 (10 *μ*M) on WDR5 and downstream EMT effectors in SW620-Scr and SW620-sh cells (*P<0.05; n.s.: not significant). (**f** and **g**) Representative images and quantification of transwell assays. Data are presented as the mean±S.D. of triplicate samples (Student's *t*-test; ***P<0.001; n.s.: not significant). (The SW620-sh#2 and SW620-sh#1 data were similar; thus, only results for SW620-sh#2 are shown.)

Western blotting revealed increased expression of phosphorylated AKT, Snail1, ZEB1, and WDR5 and decreased E-cadherin and ZO-1 expression in SW620-Scr and SW620-sh cells with IGF-1 stimulation (Figure 5a). As expected, the mRNA levels of WDR5, Snail1, and ZEB1 were markedly increased in both SW620-Scr and SW620-sh cells after IGF-1 treatment, whereas E-cadherin and ZO-1 mRNA levels were significantly downregulated under the same conditions (Figure 5d; *P*<0.05). Moreover, knockdown of WDR5 reversed the impact of IGF-1 on these molecules (Figure 5a and d; *P*<0.05). These data demonstrate that PI3K/AKT pathway activation increases WDR5 expression and then induces the dysregulation of EMT markers.

Correspondingly, when LY294002 was used to inhibit the PI3K/AKT pathway, WDR5, Snail1, and ZEB1 expression was decreased, and E-cadherin and ZO-1 were upregulated (Figure 5b).

Interestingly, when WDR5 was silenced with shRNA, the effect of LY294002 on the EMT markers was partially weakened, further strengthening the evidence that the PI3K/AKT pathway influences EMT via WDR5 (Figure 5c and e). Moreover, the transwell assays clearly showed that LY294002 inhibited the metastatic ability of CRC cells through a WDR5-dependent PI3K/AKT axis (Figure 5f and g).

These results suggest that WDR5 is elevated by PI3K/AKT pathway activation, which eventually induces EMT.

### ZNF407 is an essential downstream target of WDR5 promotion effects in CRC metastasis

**Figure 6:**
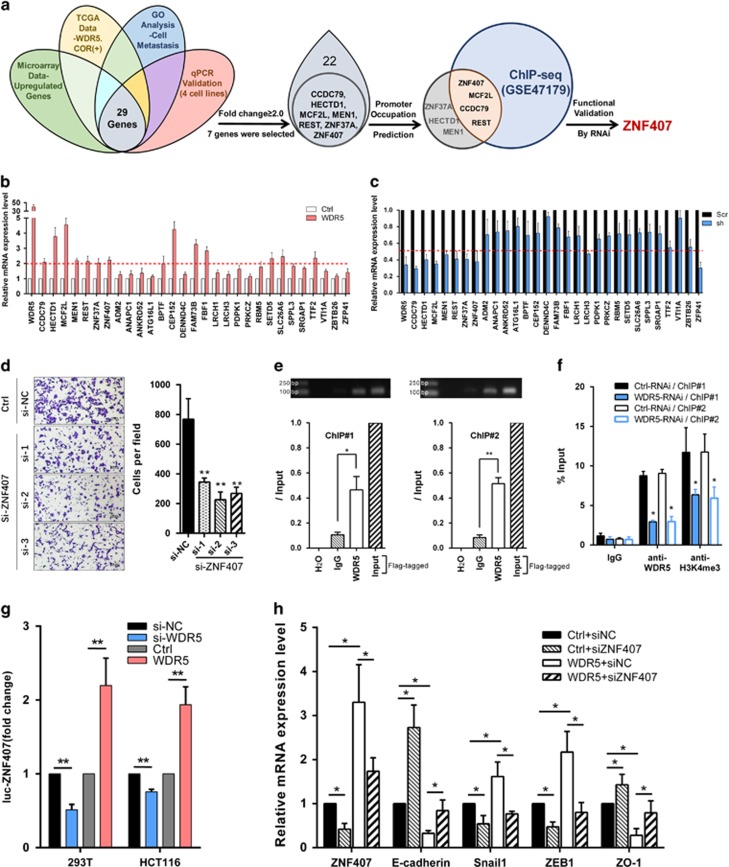
WDR5 promotes CRC metastasis by directly regulating ZNF407. (**a**) Left: Venn diagram of overlapped genes in microarray data (upregulated, fold change ≥2.0), TCGA data (positively related, *P*<0.01), gene ontology analysis (related to cell metastasis), and qPCR validated gene sets (in four CRC cell lines); middle: seven genes with fold change no less than 2.0 in qPCR validation were sorted out for further analysis; right: four candidate genes were picked out by intersecting with ChIP-seq data (GSE47179), and ZNF407 was finalized as a functional downstream target of WDR5. (**b** and **c**) Twenty-nine indicated genes were validated by qRT-PCR in WDR5-overexpressing cells (**b**) and WDR5-knockdown cells (**c**). (**d**) Representative images and quantification of transwell assays in HCT116 cells treated with ZNF407-siRNAs. Data are presented as the mean±S.D. of triplicate samples (**P<0.01). (**e**) Binding efficiency was evaluated by ChIP-PCR (upper, electrophoretogram) and ChIP-qPCR (down) using anti-Flag antibody in 293T cells with Flag-WDR5 plasmids transfected (*P<0.05; **P<0.01). (**f**) WDR5 knockdown decreases the occupancy at the ZNF407 promoter. Levels of binding efficiency was examined in indicated ChIPs using anti-WDR5 or anti-H3K4me3 antibodies, respectively. (**g**) Indicated WDR5 overexpressed/downregulated cells were transfected with ZNF407 promoter linked to luciferase, and after 24 h, luciferase activity was assayed. (**h**) mRNA expression levels of the indicated genes in HCT116 cells transfected with WDR5 and siRNA-ZNF407 (*P<0.05). Primers (ChIP#1, ChIP#2) for ChIP-PCR and ChIP-qPCR were described in Materials and Methods

Finally, to identify the downstream target genes of WDR5, gene expression profiling of HCT-15-WDR5 and HCT-15-Ctrl cells was conducted by microarray analysis. Moreover, TCGA data were downloaded from TCGA data portal to sort out genes that were positively correlated with WDR5 with *P*<0.01. Gene ontology analysis was performed to identify the unique genes that were functionally related to cell metastasis. Moreover, the overlapped genes among these three analyses were selected to be validated in four different CRC cell lines (Figure 6a, left). With these comprehensive analysies of microarray data, gene ontology, TCGA data, and qPCR validation, we identified and verified 29 genes for further exploration (Figure 6b and c). To be more exacting and rigorous, genes with a fold change in mRNA expression of at least 2.0 were selected (Figure 6a, middle) and intersected with chromatin immunoprecipitation sequencing (ChIP-seq) data (GSE47179)^29^ to predict WDR5's occupation at the promoter (Figure 6a, right; Figure 6b; Figure 6c). As a result, MCF2L, CCDC79, REST, and ZNF407 were chosen as candidates. Hence, we designed siRNAs and conducted functional assays, results revealed that no other genes but ZNF407 was picked out that can regulate the metastatic ability of CRC cells significantly (Figure 6d; Supplementary Figure S3A; *P*<0.01).

To verify the prediction of binding between WDR5 and the ZNF407 promoter, Flag-WDR5 was specially detected at the ZNF407 promoter in 293T cells by ChIP analyses (Figure 6e). Moreover, WDR5 knockdown decreased the occupancy at ZNF407 promoter using anti-WDR5 or anti-H3K4me3 antibody, indicating that WDR5 and trimethylated H3K4 are enriched at the promoter of ZNF407 (Figure 6f). Moreover, luciferase assays demonstrated that WDR5 could upregulate ZNF407 promoter activity, which further confirmed that WDR5 promotes the transcription of ZNF407 by binding to its promoter region (Figure 6g).

Furthermore, rescue assays evaluated by qRT-PCR showed that siRNA targeting ZNF407 reduced the expression levels of Snail1 and ZEB1, and significantly induced E-cadherin and ZO-1 mRNA expression; exogenous WDR5 expression could partially reverse these effects (Figure 6h). Ultimately, our results indicate that WDR5 potentially promotes CRC metastasis by targeting and stimulating the transcription of ZNF407.

Taken together, our study attempted to figure out that WDR5 is frequently overexpressed in CRC cell lines and tissues, and promotes CRC metastasis. Inhibiting WDR5 or the associated signaling pathways may improve the efficacy of CRC treatment as an effective strategy.

## Discussion

CRC development involves multiple genetic and epigenetic alterations, which results in the dysregulation of oncogenes and tumor suppressor genes.^30^ Functional loss of *PTEN,* activating mutation in *Ras* or *PIK3CA,* and stimulation by various growth factors in CRC represent mechanisms for activating the PI3K/AKT pathway. Herein, we described the tumorigenic function of WDR5 in CRC metastasis as a downstream target of the PI3K/AKT pathway, which leads to the altered expression of EMT markers and regulates metastasis by directly promoting the transcription of ZNF407. Several cell lines and animal models support our conclusion and proposed model (Supplementary Figure S4).

As reported, WDR5 is a major driver of cell progression in various cancer types. For instance, WDR5 has central roles in the proliferation of androgen-dependent prostate cancer cell, and its protein expression levels are positively correlated with poor survival in breast and bladder cancer.^15, 17, 18^ Nevertheless, none of these studies has elucidated the functional mechanism of action of WDR5 in CRC metastasis. In our study, WDR5 upregulation was frequently observed in CRC cell lines and tissues, and its overexpression level could serve as an independent predictor for survival of CRC patients. In functional studies, WDR5 significantly promoted CRC migration, invasion, and sphere formation *in vitro*, and also strengthened the ability to metastasize to the liver and lung *in vivo*. Thus, these results are in accordance with the finding that WDR5 involves in CRC metastasis, and can contribute independently as a favorable prognostic factor for OS, indicating that WDR5 can act as a potential biomarker for CRC patients.

As EMT stays crucial in tumor migration and invasion during progression and the acquisition of stemness properties,^31, 32, 33^ we tested whether WDR5 affected cell motility by inducing EMT. As expected, WDR5 overexpression decreased epithelial markers and increased mesenchymal markers at both mRNA and protein levels. Moreover, WDR5 and E-cadherin expression levels were related (*r*^2^=0.209, *P*<0.05; Pearson's test). Additional studies revealed that WDR5 was upregulated by the PI3K/AKT pathway, leading to EMT in CRC cells. Interestingly, in the absence of WDR5, EMT regulation by PI3K/AKT was seriously weakened. It has been reported that PI3K/AKT can trigger cellular EMT.^34^ Our data present an explanation for how WDR5 is modulated in CRC and confirm that the PI3K/AKT pathway-induced EMT entails WDR5 profoundly. More importantly, these findings provide fresh possibilities for molecular intervention in CRC as a target or as a cooperative factor to enhance the efficacy for molecule-targeting agent.

Furthermore, microarray assays were performed, and gene expression data were analyzed using various databases (i.e., gene ontology, GEO, and TCGA). Eventually, ZNF407 was determined to be directly targeted by WDR5, and CRC cell metastasis was inhibited when ZNF407 was silenced. Additional data revealed that knockdown of ZNF407 partially rescued the WDR5-induced decrease in E-cadherin mRNA expression (Figure 6h). Moreover, it was reported that missense mutations in ZNF407 affect tumor progression, as evidenced by whole-genome sequencing of gastrointestinal stromal tumors.^28^ Moreover, our functional studies of ZNF407 showed that ZNF407 functioned as a promotive factor in CRC metastasis and accelerated cell proliferation slightly (Supplementary Figure S3). Together, these data suggest a novel theory that CRC metastasis is regulated by WDR5.

Although our studies led to these important discoveries, there are some limitations. First, we detected a slight, but not significant promotion of CRC cell growth in MTT and colony formation assays (data not shown). We tentatively suggest that WDR5 functions in stemness or the maintenance of cell viability, not growth, which is in conformance with the results in Figure 2c and d, showing more but not bigger spheres in the WDR5 overexpressed group than that in the control group. Notably, it is reported that tumor cells stop dividing when they undergo EMT; hence, EMT acts as a suppressor of cancer cell division, thus stagnating proliferation.^35, 36^ Therefore, WDR5 might be more appropriately categorized as a metastasis promoter than a tumor growth inducer. Second, CRC predominantly metastasizes to the liver and lung;^37, 38^ we designed two *in vivo* models to show that WDR5 has a positive effect on CRC metastasis. Importantly, in the multistep metastatic process, cancer cells must first survive in the circulation system and then implant at a distant organ site. On the basis of this phenomenon, *in vitro* sphere formation assays were conducted, and a higher sphere formation efficiency was observed in cells overexpressing WDR5 that were cultured ultralow attachment plates. In addition, Cox correlation analysis of CRC tissues indicated that WDR5 is an independent prognostic factor for PFS. Hence, our results imply that WDR5 takes pivotal part in CRC metastasis process. Third, WDR5 was reported to interact with HDAC3, resulting in a recruitment of a histone methyltransferase complex and the activation of mesenchymal gene expression under hypoxic conditions.^39, 40, 41^ Indeed, qRT-PCR analysis of CoCl_2_-treated CRC cells showed a dose-dependent activation of HIF1-α, followed by the upregulation of WDR5 expression (data not shown). These findings suggest another potential mechanism by which WDR5 is elevated in CRC and provide reliable data to explain how WDR5 regulates EMT. Fourth, the role of EMT in metastasis is not yet completely understood, and some studies suggest that EMT is not the speed-limiting process for metastasis, instead it highlights the importance of EMT-induced chemoresistance.^35, 42^ Furthermore, accumulating evidence indicates that cancer stem cells are characteristically resistant to conventional cancer therapy and drive tumor progression, metastasis, and chemoresistance.^43^ A previous study reported that PI3K/AKT induced chemoresistance in colon cancer cells,^44^ coinciding with the regulation of cellular self-renewal and the reprogramming with a core transcription network by WDR5, which was found to be activated by PI3K/AKT to promote CRC metastasis in our study. The role and mechanism of action of WDR5 in CRC chemoresistance represents a future research direction.

In conclusion, WDR5 is vital for cell development, and its dysregulation is essential in CRC particularly. We demonstrate that WDR5 is upregulated in CRC and promotes CRC metastasis by clarifying the underlying mechanism. This study show that WDR5 is a valuable marker for CRC and has a pivotal role in CRC metastasis.

## Materials and Methods

### Patient tissue specimens and follow-up

All formalin-fixed, paraffin-embedded colorectal tissues used in this study were archived from Sun Yat-sen University Cancer Center (SYSUCC) with written informed consent following the Ethics Committee approval, and associated clinical data and pathology report review were obtained from raw case reports.

Each tissue sample was collected from patients before surgery and neoadjuvant therapy from 1 September 1999 to 31 December 2007, containing tumor tissues (percentage of tumor cells >70%) and non-tumor tissue (>5 cm lateral from the cancerous edge). All patients were contacted for at least 5 years, and the last censor date was 1 March 2014.

The normalized level_1 clinical data and mRNAseq data are from TCGA database (data version 2016_01_28) for the Colorectal Adenocarcinoma (COADREAD) project.

### Cell lines, cell culture, and stimulation

The HEK-293T (human embryonic kidney cell line), FHC (normal colon epithelium cell line), and the human CRC cell lines, including HCT116, HT-29, HCT-15, SW620, and COLO205, were obtained from American Type Culture Collection, and cultured under standard conditions. GP293 cells were from our own laboratory and grown in DMEM.^45^ All cells were regularly authenticated by STR profiling at the China Center for Type Culture Collection. Sensitive Mycoplasma detection was conducted regularly by a PCR-based method.

For blockade or activation of the PI3K/AKT pathway, LY294002 (Cell Signaling Technology, Boston, MA, USA) or IGF-1 (Cell Signaling Technology) was used. Cells were plated in six-well plates, and 24 h after seeding, cells were treated for another 24 h with LY294002 (10 *μ*M), IGF-1 (100 ng/ml), or DMSO/PBS (control) in a serum-free medium. Cells were then washed in PBS, RNA was isolated for qRT-PCR, and cell lysis was used in western blot analysis as described below.

### Quantitative reverse transcription PCR

Total RNA was extracted from cultured cells using TRIzol (Invitrogen, Carlsbad, CA, USA), and was reverse-transcribed using M-MLV reverse transcriptase (Promega, Madison, WI, USA). cDNA was used as template for quantitative reverse transcription PCR (qRT-PCR) using Stratagene MX3000P Sequence Detection System with SYBR Green qPCR mixture (Invitrogen). Each qRT-PCR experiment was performed in triplicate. GAPDH was used as loading control. The primer sequences of the indicated genes are provided in Supplementary Table S3.

### Immunohistochemical staining

IHC staining was performed according to a previous study.^45^ Briefly, IHC for WDR5 was performed in 161 paraffin-embedded primary CRC tissues and adjacent noncancerous tissues. The following primary antibodies were used: rabbit anti-E-cadherin monoclonal antibody (1:200, Cell Signaling Technology) and goat anti-WDR5 polyclonal antibody (1:100, R&D System Inc, Minneapolis, USA). Antibody dilution solution was used as negative control. IHC staining was evaluated using the H-score and dichotomized according to OS with an ROC curve.

### Western blot analysis

Whole-cell lysates were obtained by RIPA lysis buffer. Primary antibodies against GAPDH (1 : 1000, Santa Cruz Biotechnology, Santa Cruz, CA, USA), E-cadherin, Snail1, ZEB1, ZO-1, PI3K, AKT, phospho-AKT (1:1000, Cell Signaling Technology), and WDR5 (1:500, R&D) were used. Enhanced chemiluminescence (Perkin Elmer, Hopkinton, MA, USA) was used to visualize the protein bands transferred to the PVDF membranes. Detailed standard western blotting was performed as described previously.^46^

### Cell transfection

Full-length cDNA corresponding to human WDR5 (primers: forward: 5′-CCCAAGCTTCATGGCGACGGAGGAGAAG-3′ reverse: 5′-CCGGAATTCTTAGCAGTCACTCTTCCACAG-3′) was inserted into the pcDNA3.1 and pCMV-N-Flag vectors. All siRNAs were purchased from GenePharma, and the siRNA sequences are shown in Supplementary Table S2. A negative siRNA control was used. Each resulting construct and sequence was confirmed. To perform transient transfections, HCT116 and HT-29 cells were incubated at 3–5 × 10^5^ cells/well in six-well plates at a confluence of 50–60%. Twenty-four hours later, plasmid DNA (4 *μ*g) or siRNA (100 pmol) was transfected into cells following the manufacturer's protocol using Lipofectamine 2000 (Invitrogen).

### Stable cell line construction

WDR5 full-length sequence and control sequence were inserted into the pLNCX2 viral vector (Clontech, Mountain View, CA, USA). WDR5 shRNA was synthesized at Invitrogen and the short hairpin target sequences are provided in Supplementary Table S2. Stable cell lines HCT-15 for overexpressing WDR5 and SW620 for silencing WDR5 were constructed by lentivirus packaging and transduction according to a previous method with slice modifications.^45^ WDR5 expression in these stable cell lines was validated by western blotting and qRT-PCR evaluation.

### Migration and invasion assays

Tumor cells (1 × 10^5^ each) were serum-starved and seeded into Boyden chambers with or without Matrigel-coated inserts (8-*μ*m pore, BD Falcon, Franlin Lakes, NJ, USA) for invasion or migration assays, respectively. The chambers were then placed in 24-well plates containing medium and 10% FBS. After 24 h, cells attached to the undersurface of the membrane were fixed in ethanol, stained with crystal violet, and counted using a microscope.

### Sphere formation assay

Detailed protocol was described previously.^47, 48^ Briefly, a single-cell suspension of tumor cells was plated in ultralow attachment plates (Corning, Tewksbury, NY, USA) in serum-free DMEM supplemented with human recombinant epidermal growth factor (20 ng/ml, Invitrogen) at a density of 5000 cells/well. Spheres larger than 50 *μ*m were counted after 1–2 weeks.

### *In vivo* metastasis assay

Animal studies were performed according to the Chinese standards and regulations, and approved by the committee of Sun Yat-sen University Cancer Center. Nude mice (BALB/c, female, 4–5 weeks of age, 15–18 g) were purchased from the Slaccas Experimental Animal Center (Shanghai, China).

We chose the HCT-15 and SW620 stable cell lines for the hepatic and lung metastasis models, respectively, based on preliminary experiments.

Hepatic and lung metastasis models were used.^49^ Briefly, 1 × 10^6^ cells (HCT-15-Ctrl, HCT-15-WDR5, SW620-Scramble, or SW620-shWDR5#2) were injected into the distal tip of the spleen or the lateral tail vein using an insulin syringe, and allowed to grow for 2 months. Euthanasia was conducted after injection, and the number of nodule on the liver and lung surfaces was counted. Livers and lungs were formalin-fixed for further evaluation.

### Microarray analysis

Total RNA was isolated as described above. After undergoing quality control, the samples were scanned using an Agilent 44 K Whole Human Genome Microarray by Shanghai Biotechnology Corporation (Sanghai, China). Differential gene expression was determined using the Gene Spring Software (Santa Clara, CA, USA), and gene ontology analysis was performed.

### Chromatin immunoprecipitation

ChIP was carried out according to the manufacturer's instruction of the ChIP Assay Kit (Beyotime, Shanghai, China). The pCMV-Flag-WDR5 plasmid was transfected into cells. Anti-Flag (Sigma-Aldrich, St. Louis, MO, USA), anti-H3K4me4 (Abcam, Cambridge, MA, USA), and anti-WDR5 (R&D) antibodies were used, and real-time qPCR was performed in a Stratagene MX3000P Sequence Detection System. These two primers were used to amplify the ZNF407 promoter:

ChIP#1: forward: 5′-CTTCAGGCAGCACACGGTA-3′, reverse: 5′-GGTGAAAGTGCTGCTTCGTC-3′

ChIP#2: forward: 5′-CTGAAGTCCAGGGTAAGCCA-3′, reverse: 5′-AGTCTTGACCATGCCGAAGC-3′.

### Luciferase assay

This was carried out as the manufacturer's instructions (Promega). Briefly, 4 × 10^4^ cells per wells were plated in 24-well plates and transfected with 500 ng ZNF407 promoter-luciferase plasmids (Generay Biotechnology, Shanghai, China) and pcDNA3.1-WDR5 (500 ng) or siRNA-WDR5 (100 pmol). To normalize the efficiency, cells were co-transfected with 10 ng TK. Twenty-four hours later, the luciferase activity was measured using the Dual-luciferase assay kit (Promega).

### Statistical analysis

Statistical analysis was performed using the version 17.0 SPSS software (SPSS Inc, Chicago, IL, USA). All the *in vitro* and *in vivo* experiments were repeated for three times at least, and one representative data are presented. Association between WDR5 expression and clinicopathological parameters was evaluated by two-tailed *χ*^2^-tests. Pearson's test was applied for the correlation analysis. OS and DFS were analyzed with Kaplan–Meier survival and log-rank test. A Cox regression model was used for multivariate survival analysis. The significance between subgroups was compared using the two-tailed Student's *t*-test. *P*<0.05 was considered statistically significant.

## Figures and Tables

**Table 1:** Clinicopathological variables and correlations with WDR5 expression in 161 cases of CRC

**Variables**	**All cases (*****N*****=161; %)**	**WDR5**		***P*****-values**
		**Low expression (*****n*****=63; %)**	**High expression (*****n*****=98; %)**	
*Gender*				
Male	92 (57.1)	34 (21.1)	58 (36.0)	0.517
Female	69 (42.9)	29 (18.0)	40 (24.9)	
				
*Age, year*				
<65	118 (73.3)	46 (28.5)	72 (44.8)	0.95
≥65	43 (26.7)	17 (10.6)	26 (16.1)	
				
*Location*				
Colon	83 (51.6)	33 (20.5)	50 (31.1)	0.867
Rectum	78 (48.4)	30 (18.6)	48 (29.8)	
				
*Size, cm*				
<5	59 (36.6)	26 (16.1)	33 (20.5)	0.332
≥5	102 (63.4)	37 (23.0)	65 (40.4)	
				
*Grade*				
Poor	40 (24.8)	9 (5.6)	31 (19.3)	0.54
Moderate	121 (75.2)	54 (33.5)	67 (41.6)	
				
*TNM stage*				
I–II	90 (55.9)	45 (27.9)	45 (28.0)	0.001^b^
III–IV	71 (44.1)	18 (11.2)	53 (32.9)	
				
*Lymph node status*				
0	108 (67.1)	52 (32.3)	56 (34.8)	0.001^b^
≥1	53 (32.9)	11 (6.8)	42 (26.1)	
				
*Distant metastasis*				
M0	116 (72.0)	51 (31.7)	65 (40.4)	0.044^b^
M1	45 (30.0)	12 (7.5)	33 (20.5)	
				
*CEA,*^a^ *ng/ml*				
<5	76 (67.9)	32 (28.6)	44 (39.3)	0.614
≥5	36 (32.1)	17 (15.2)	19 (17.0)	

NOTE: The numbers in parentheses indicate the percentages of tumors with a special clinical or pathologic feature for a given WDR5 subtype

a Analysis of this parameter was available for 112 cases

b Statistically significant

**Table 2:** Multivariate analysis for PFS and OS

**Variables**	**PFS, HR (95% CI)**	***P***-**value**	**OS, HR (95% CI)**	***P*** **-value**
TNM stage (I–II versus III–IV)	0.477 (0.174–1.304)	0.149	0.545 (0.192–1.543)	0.253
Lymph node status (0 versus ≥1)	1.101 (0.573–2.116)	0.772	0.893 (0.448–1.781)	0.747
Distant metastasis (no versus yes)	0.177 (0.082–0.383)	<0.01^a^	0.144 (0.066–0.312)	<0.01^a^
WDR5 (low versus high)	0.574 (0.336–0.979)	<0.05^a^	0.573 (0.326–0.999)	<0.05^a^

Abbreviations: CI, confidence interval; HR, hazard ratio; OS, overall survival; PFS, progression-free survival

a Statistically significant
