# Supplementary figures and images for: PI3K/AKT-mediated upregulation of WDR5 promotes colorectal cancer metastasis by directly targeting ZNF407
Source: Cell Death Dis. 2017 Mar 16;8(3):e2686–. doi: 10.1038/cddis.2017.111 (PMC5386518; doi:10.1038/cddis.2017.111)

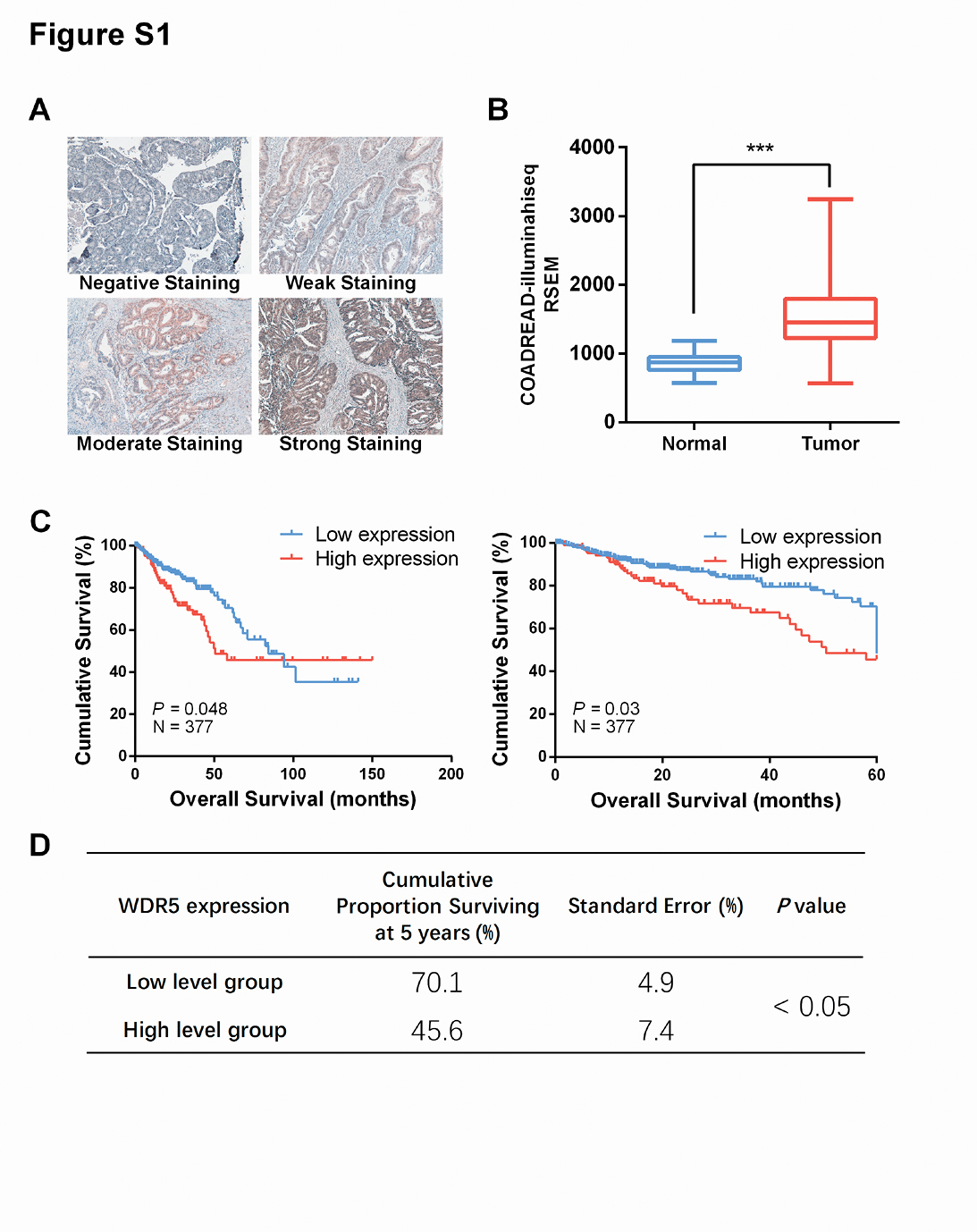

Supplement: Supplementary Figure S1 [file cddis2017111x1.tif]

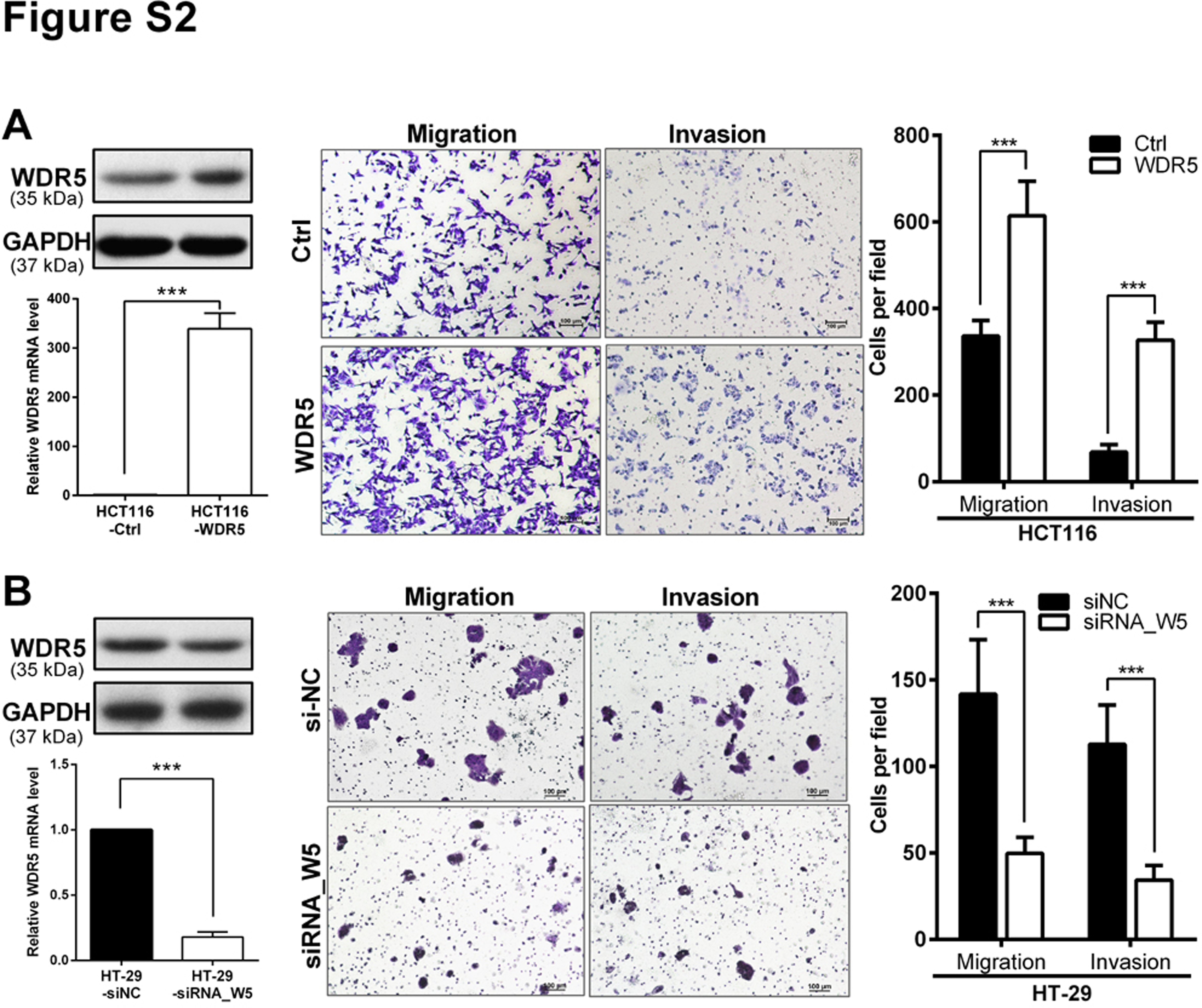

Supplement: Supplementary Figure S2 [file cddis2017111x2.tif]

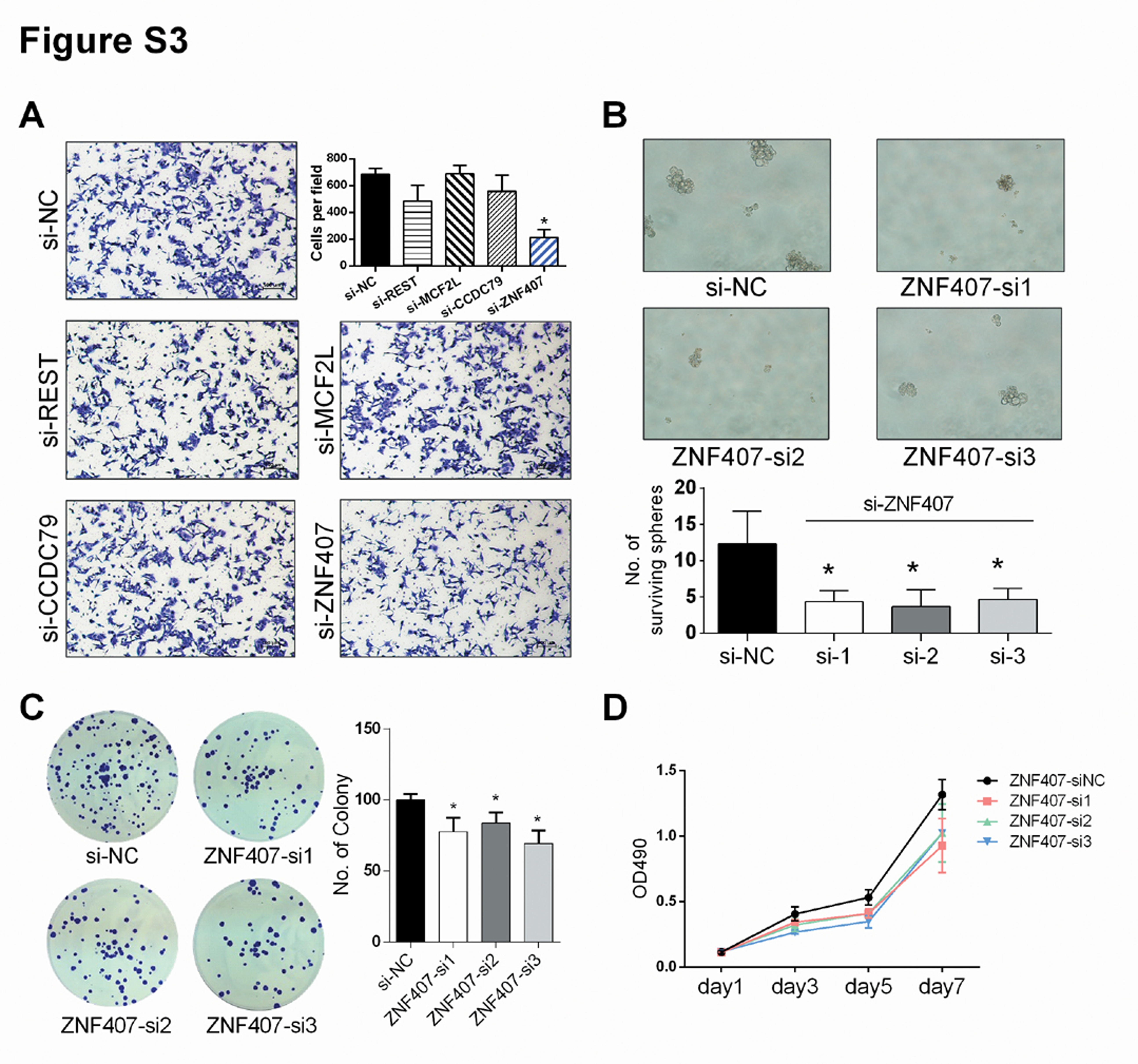

Supplement: Supplementary Figure S3 [file cddis2017111x3.tif]

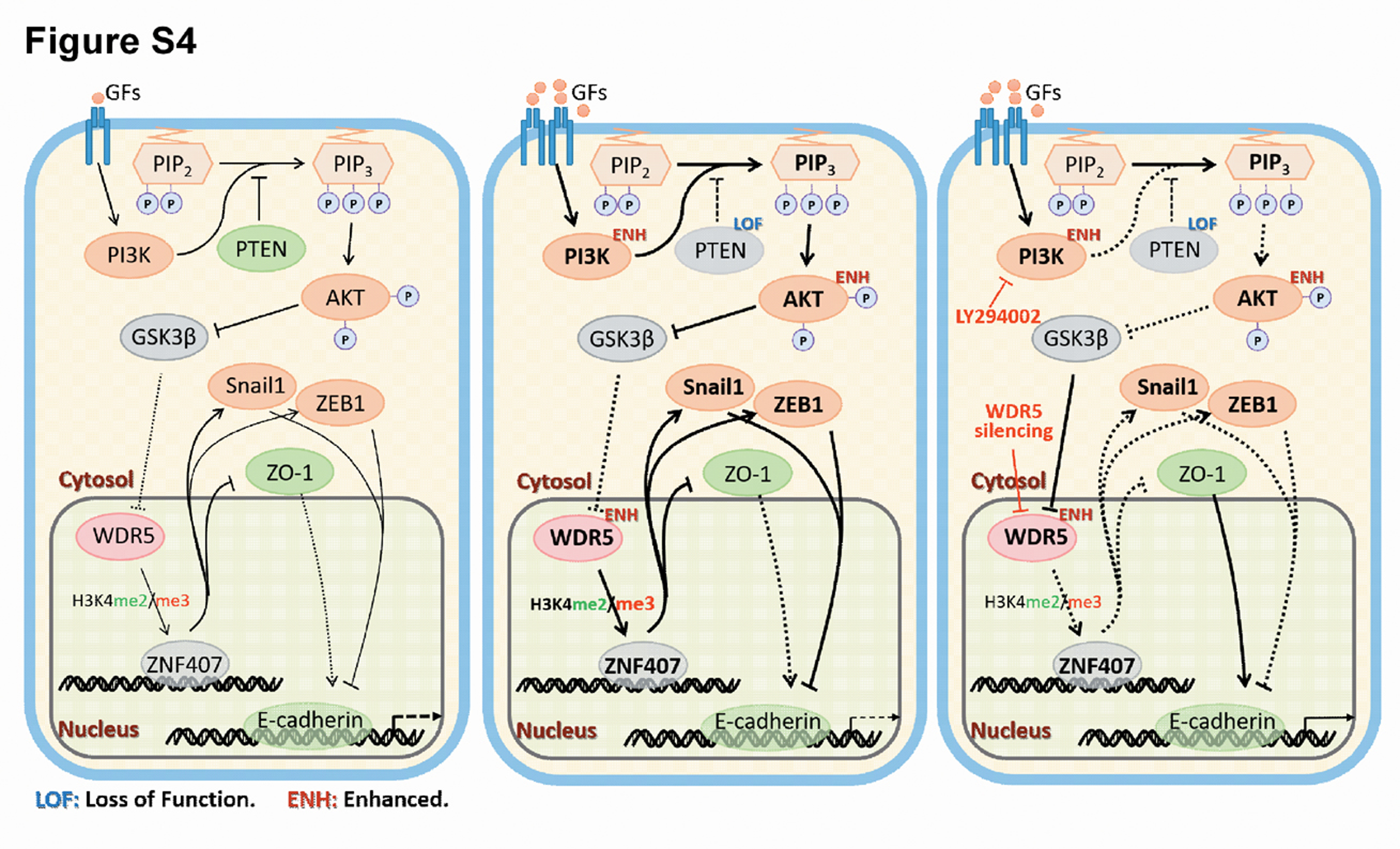

Supplement: Supplementary Figure S4 [file cddis2017111x4.tif]
